# [Corrigendum] Neuroprotective effect of emodin against Alzheimer’s disease via Nrf2 signaling in U251 cells and APP/PS1 mice

**DOI:** 10.3892/mmr.2023.12994

**Published:** 2023-04-05

**Authors:** Zhiping Li, Hui Bi, Hongbo Jiang, Jingjing Song, Qingfan Meng, Yizhi Zhang, Xiaofang Fei

Mol Med Rep 23: 108, 2021; DOI: 10.3892/mmr.2020.11747

Subsequently to the publication of this paper, an interested reader drew to the authors’ attention that the lower left panel of Fig. 3A of this paper had already featured in the following paper, which featured one of the same authors (Zhiping Li): Zhang Y, Wang J, Wang C, Li Z, Liu X, Zhang J, Lu J and Wang D: Pharmacological basis for the use of evodiamine in Alzheimer's disease: antioxidation and antiapoptosis. Int J Mol Sci 21: 1527, 2018. Moreover, an independent analysis of the data in this paper conducted by the Editorial Office revealed that the Bcl-2 protein western blotting data featured in Fig. 3C had apparently also appeared in a previous publication featuring the same author [Qiu Y, Jiang X, Liu D, Deng Z, Hu W, Li Z and Li Y: The hypoglycemic and renal protection properties of crocin via oxidative stress-regulated NF-κB signaling in db/db mice. Front Pharmacol 30: 541, 2020].

After having examined their original data, the authors have realized that Fig. 3 in the above paper had been inadvertently assembled incorrectly, owing to the mishandling of certain of the data. In addition, the authors wished to present a revised version of Fig. 4 containing more representative data for Fig. 4C and D.

The corrected versions of Figs. 3 and 4, featuring the correct data for Fig. 3A and C, and the revised data in Fig. 4C and D, are shown on the next two pages. Note that these errors did not significantly affect the results or the conclusions reported in this paper, and all the authors agree to the publication of this Corrigendum. The authors are grateful to the Editor of *Molecular Medicine Reports* for granting them the opportunity to publish this corrigendum, and apologize to the readership for any inconvenience caused.

## Figures and Tables

**Figure 3.**
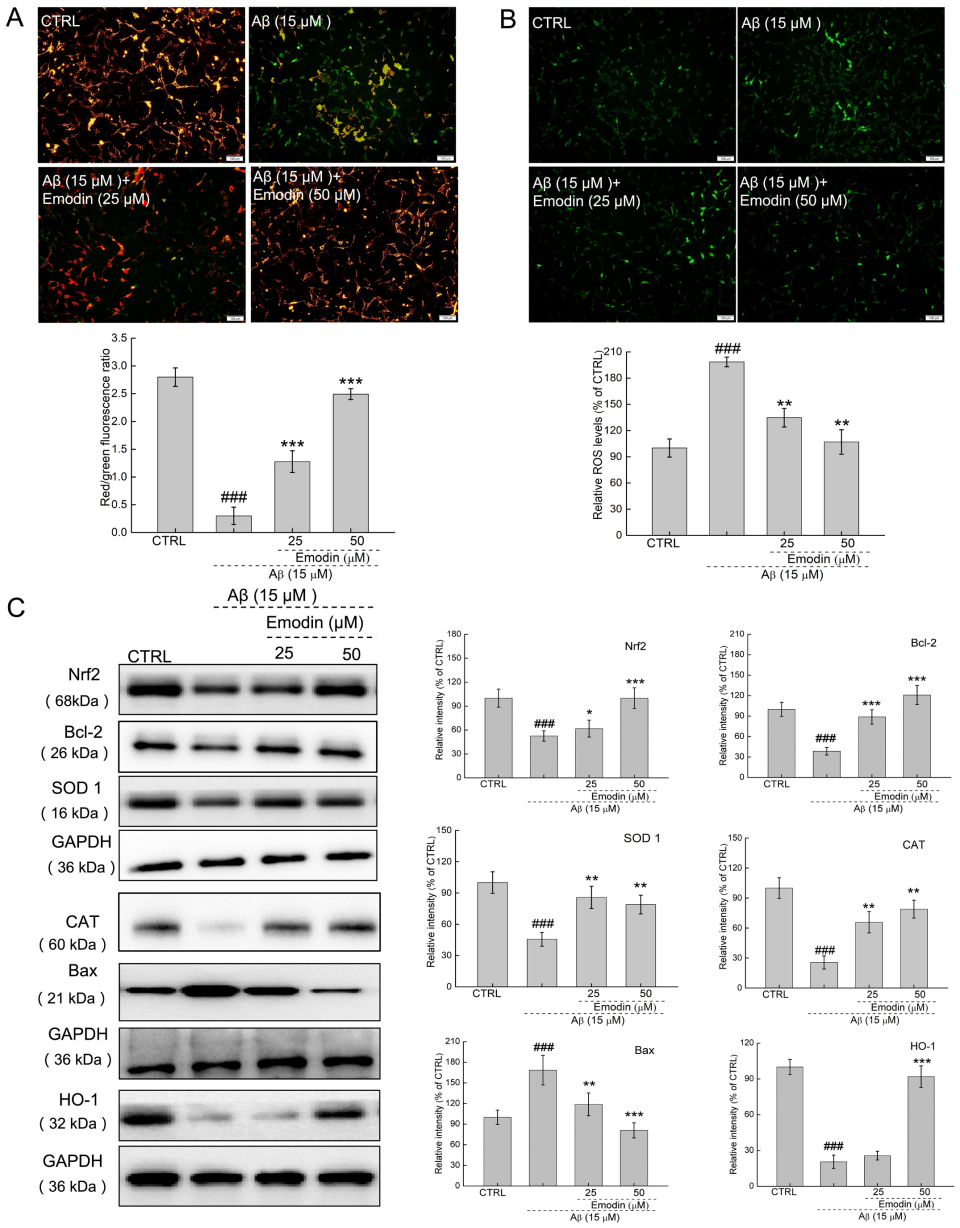
Emodin suppresses oxidative stress by increasing the expression levels of Nrf2 in U251 cells undergoing Aβ1-42-induced apoptosis. A 3-h emodin pre-treatment (A) ameliorated dissipation of mitochondrial membrane potential and (B) inhibited excessive ROS accumulation in U251 cells exposed to Aβ for 24 h (magnification, ×10; scale bar, 100 µm). (C) Levels of oxidative stress- and apoptosis-associated proteins in Aβ1-42-exposed U251 cells were examined by western blotting. Quantitative protein expression levels were normalised to those of GAPDH. Data are presented as the mean ± SEM (n=6 experiments). ^###^P<0.001 vs. CTRL. *P<0.05, **P<0.01 and ***P<0.001 vs. Aβ1-42 only. Nrf2, nuclear factor E2-related factor 2; Aβ, β-amyloid peptide; ROS, reactive oxygen species; CTRL, control; SOD, superoxide dismutase; CAT, catalase; HO, heme oxygenase.

**Figure 4.**
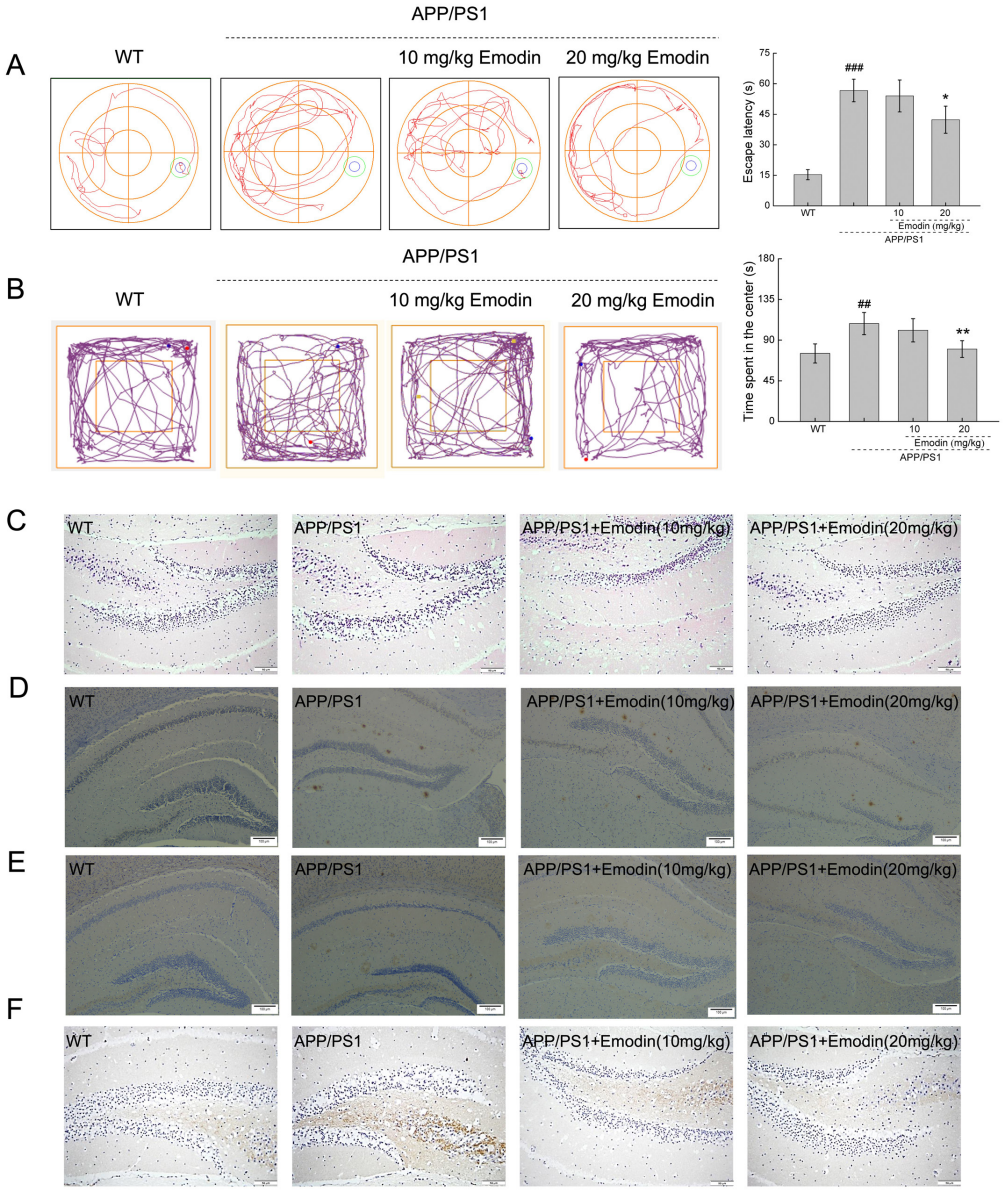
Emodin improves behavioral performance of APP/PS1 mice by decreasing deposition of Aβ1-42, p-τ and 4-HNE. (A) Emodin decreased the escape latency time of APP/PS1 mice during the Morris water maze test. (B) Emodin decreased the time spent by APP/PS1 mice in the central area during the open field test. Data are presented as the mean ± SEM (n=10). ^##^P<0.01 and ^###^P<0.001 vs. WT mice. *P<0.05 and **P<0.01 vs. untreated APP/PS1 mice. (C) Haematoxylin and eosin staining of brain tissue (scale bar, 50 µm; n=5 experiments). (D) Emodin markedly suppressed deposition of Aβ1-42. (E) Overaccumulation of p-τ in the brain of APP/PS1 mice (scale bar, 100 µm; n=5 experiments). (F) High expression levels of 4-HNE (scale bar, 50 µm; n=5 experiments) in the brain of APP/PS1 mice detected by immunohistochemistry. APP, amyloid precursor protein; PS1, presenilin-1; Aβ, β-amyloid peptide; p-, phosphorylated; 4-HNE, 4-hydroxy-2-nonenal; WT, wild-type.

